# Intelligence

**Published:** 1832-09

**Authors:** 


### INTELLIGENCE.

### ROYAL COLLEGE OF SURGEONS. JACKSONIAN PRIZE.

THE prize subject for 1833 is, Formation, Constituents, Extraction, of Urinary Calculi. Candidates to be members of the College, not of the Council. Dissertations to be addressed to the Secretary, and delivered at the College, before Christmas-day, 1833.

The prize subjects for the preseut year, 1832, are, the symptoms occasioned by the different Mineral Poisons, when taken into the Stomach; the proper Antidotes and Treatment which experience or science affords for each; and, when the event is fatal, the appearance or effects which are generally found in the Dead Body: and the Mode of Union of Simple and Compound Fractures. Dissertations upon which must be delivered at the College before Christmas-day next.

EDMUND BALFOUR, Secretary.

### KING'S COLLEGE, LONDON.

THE Medical School in King's College will open on the 1st of October; the introductory lecture to be delivered at three P.M., by Professor GREEN.

The introductory lecture to the Chemical Course will be delivered by Professor DANIELL, on Tuesday, October 2d, at nine A.M.

The introductory lecture to the Anatomical Course, on Tuesday, at three P.M., by Professor MAYO.

And the Introductory Lecture to the Botanical Courses, on Wednesday, the 3d, at three P.M., by Professor BURNETT: the popular course to be continued every Wednesday, at the same hour; the scholastic course every morning, at eight.

The family of the late Dr. SIMS have liberally presented to the Museum of King's College, London, the whole of that gentleman's very valuable Herbarium, containing the original specimens from which the drawings for the figures in the Botanical Magazine were made.

LONDON HOSPITAL.

Dr. A. FRAMPTON has been elected Physician to the London Hospital, in place of Dr. MACBRAINE, who has resigned.

---

THE CHOLERA AND THE LIFE INSURANCE COMPANIES.

It was recently stated, at a meeting of the various Insurance Companies in London, that, since the first appearance of cholera in the United Kingdom, up to the 27th ultimo, there had been only thirty-six claims on account of deaths occasioned by this disease. This fact was adduced as a proof that the ravages of the disease have been chiefly confined to the lower orders of society.

---

*Cholera Act.* A surgeon of Warrington, of the name of Greirson, was recently convicted in the full penalty of 5*l.*, for refusing to return a case of cholera to the Local Board of Health. As Mr. G. promised not to withhold his returns in future, he was proceeded against for one case only.

---

*Anatomical Inspectors.* Dr. JAMES SOMERVILLE has been appointed inspector for England, and Dr. CRAIGIE, of Edinburgh, inspector of Scotland, under the new Anatomical Bill.

---

THE CHOLERA.

*Council Office, Whitehall; August* 29.

Remaining at last report, 2,749; new cases, 1,051; died, 335; recovered, 611; remaining, 2,854; total cases from commencement, 42,222; total deaths from commencement, 15,451.

---

CENTRAL BOARD OF HEALTH FOR IRELAND.

*Council Office, Dublin Castle; August* 17.

Remaining at last report, 359; new cases, 33; died, 5; recovered, 37; remaining, 350; total cases from commencement, 9,676; total deaths from commencement, 2,758.

### LITERARY INTELLIGENCE.

In the press, and will be published on the 1st of October, (to be continued monthly,) No. I. of a new edition of "Medical Botany;" edited by GILBERT T. BURNETT, F.L.S. &c., Professor of Botany in King's College, London. Also, by the same author, is preparing for the press, and shortly will be published, "A Practical Guide to the Study of Plants," designed chiefly for the use of medical students.

*Morbid Anatomy.* Dr. CARSWELL is about to publish "Elements of Morbid Anatomy," illustrated by selections from his numerous and valuable drawings. Dr. C. has very long been engaged upon the subject of morbid anatomy, and we have no doubt his work will be such as to merit the attention of the profession.

### METEOROLOGICAL JOURNAL,

By Messrs. HARRIS and Co. Mathematical Instrument Makers, 50, High Holborn.

| | Rain gauge. | Moon. | Thermometer. | | | Barometer. | | De Luc's Hygrometer. | | Winds. | | Atmospheric Variation. | | |
|---|---|---|---|---|---|---|---|---|---|---|---|---|---|---|
| *July* | | | *9a.m.* | *max.* | *min.* | *9a.m.* | *10p.m.* | *9a.m.* | *10p.m.* | *9 a.m.* | *10p.m.* | *9 a. m.* | *2 p.m.* | *10 p. m.* |
| 20 | | ☾ | 63 | 65 | 62 | 29.95 | 29.90 | 45 | 45 | N | ENE | fine | fine | cloudy |
| 21 | | | 60 | 65 | 50 | .90 | .92 | 45 | 46 | ENE | N | | | |
| 22 | | | 57 | 63 | 52 | .92 | .93 | 48 | 50 | NE | N | | | fine |
| 23 | | | 60 | 64 | 56 | .95 | .90 | 50 | 50 | NNW | W | | | cloudy |
| 24 | | | 64 | 68 | 60 | .94 | .98 | 50 | 49 | N | NNE | | | cloudy |
| 25 | | | 66 | 71 | 58 | .93 | .91 | 49 | 50 | NW | N | | | |
| 26 | | | 64 | 67 | 56 | .91 | .88 | 50 | 50 | NE | E | | | fine |
| 27 | | ● | 64 | 68 | 53 | .88 | .90 | 50 | 50 | ENE | NE | cloudy | | |
| 28 | | | 61 | 68 | 51 | .94 | 30.02 | 48 | 45 | NNE | NE | fine | | |
| 29 | | | 57 | 62 | 56 | 30.08 | .13 | 45 | 48 | NE | NE | | | |
| 30 | | | 62 | 66 | 55 | .13 | .13 | 50 | 50 | SE | SE | | | |
| 31 | | | 60 | 65 | 54 | .06 | 29.94 | 50 | 50 | SE | ESE | cloudy | | |
| *Aug.* 1 | | | 63 | 65 | 55 | 29.83 | .74 | 50 | 57 | SE | E | fine | | rain |
| 2 | .19 | | 64 | 67 | 61 | .63 | .60 | 63 | 64 | E | E | cloudy | | rain |
| 3 | .35 | ☽ | 65 | 70 | 59 | .68 | .74 | 64 | 61 | W | N | | | cloudy |
| 4 | | | 66 | 67 | 55 | .83 | .82 | 59 | 55 | NW | SW | | | fine |
| 5 | | | 63 | 68 | 59 | .70 | .62 | 57 | 61 | SW | W | rain | rain | rain |
| 6 | | | 63 | 71 | 60 | .66 | .67 | 60 | 57 | W | WSW | fine | fine | cloudy |
| 7 | .53 | | 67 | 72 | 59 | .70 | .74 | 56 | 55 | W | WNW | cloudy | | fine |
| 8 | | | 65 | 71 | 61 | .82 | .82 | 53 | 51 | SW | SSW | fine | | |
| 9 | | | 70 | 76 | 65 | .82 | .83 | 53 | 48 | SSE | ESE | | | |
| 10 | | | 72 | 79 | 68 | .89 | .96 | 47 | 48 | WNW | SW | | | |
| 11 | | ○ | 71 | 72 | 63 | 30.06 | 30.06 | 50 | 46 | SW | WSW | | | |
| 12 | | | 68 | 75 | 60 | 29.98 | 29.87 | 46 | 48 | WSW | W | | | |
| 13 | | | 64 | 68 | 59 | .70 | .65 | 47 | 49 | NE | ENE | rain | | |
| 14 | | | 65 | 70 | 64 | .65 | .68 | 50 | 51 | ENE | ESE | fine | | |
| 15 | | | 67 | 71 | 56 | .64 | .65 | 53 | 53 | S | SW | cloudy | | |
| 16 | | | 63 | 69 | 59 | .72 | .82 | 51 | 49 | W *va.* | W *va.* | fine | | |
| 17 | | | 68 | 70 | 59 | .87 | .84 | 49 | 50 | WSW | SSW | | | |
| 18 | | | 64 | 70 | 59 | .68 | .63 | 50 | 50 | SW | SW | cloudy | | rain |
| 19 | .04 | ☾ | 63 | 67 | 55 | .74 | .82 | 51 | 51 | SW | SW | rain | cloudy | fine |

The quantity of Rain fallen in the month of July, was 1 inch.

